# Patterns in fish naming ability in two fishing communities of Myanmar

**DOI:** 10.1186/s13002-023-00610-7

**Published:** 2023-08-31

**Authors:** Aung Si, Aung Kyawphyo

**Affiliations:** 1 Institute for Linguistics, University of Cologne, Albertus-Magnus-Platz, 50923 Cologne, Germany; 2 Kyaukphyu, Myanmar

**Keywords:** Burma, Ethnoichthyology, Shan state, Rakhine, Nomenclature, Artisanal fishermen, Fish seller, Language documentation

## Abstract

**Background:**

To date, there is little reliable information on the fish names used by two fishing communities of Myanmar, namely Intha (Inle Lake) and Rakhine (Bay of Bengal). Moreover, there have been no systematic studies on the distribution of fish-related traditional knowledge in these two communities. As there can be high levels of intra-community variation in traditional ecological knowledge, it is important to investigate this variation along the lines of key social variables.

**Methods:**

Fieldwork was carried out in both communities and involved the presentation of visual stimuli (colour pictures of locally relevant fish species) to respondents, and asking for a name in the local language. The stimuli consisted of 43 and 218 fish species for Intha and Rakhine, respectively. The responses were analysed in terms of respondent age and occupation for both communities, plus village location for Intha and gender whenever both genders were represented in a sufficiently large number in the sample.

**Results:**

Fish name lists were generated for both languages, taking into account lexical variation, as well as the number of people able to name each fish. The two communities differed markedly in the way fish knowledge was distributed. Overall, younger Intha knew fewer fish names, but there was little to no difference in fish knowledge among the Intha on the basis of occupation, location or gender. In contrast, the differences were very marked among Rakhine respondents.

**Conclusions:**

The reduced fish knowledge of younger Intha may be ascribed to environmental disturbances that have caused many fish to go locally extinct. The otherwise homogenous distribution of fish knowledge in the Intha community may be due to the small number of species that people are required to learn. This idea needs to be tested with a larger sample of respondents. Among the Rakhine, a number of factors are responsible for the observed variation; these include a steep learning curve among younger fishermen, the difference in fish species encountered by fishermen and sellers, highly variable dietary preferences among the general populace and differing gender roles in the context of market visits. The authors are in full agreement with previous research that advocates a variationist approach to the study of traditional ecological knowledge.

## Background

It is widely recognised that artisanal fishermen and fisherwomen possess much ecological knowledge that could be of great value to marine conservation and fisheries management [1–5]. A prerequisite first step towards a comprehensive understanding of fishermen’s ecological knowledge of the local marine environment and species is an adequate documentation of vernacular fish names. This is particularly important in the case of understudied language communities, where correspondences between vernacular names and biological fish species may be obscured by phenomena such as dialectal variation, borrowing of words from neighbouring languages and local (linguistic) innovation [6–9].

A further question of interest is the distribution of ethnobiological knowledge in a language community, which can help explain patterns of horizontal and vertical knowledge transmission in that community. It has been repeatedly demonstrated in numerous communities that the normal distribution of traditional ecological knowledge (TEK) is not uniform, but prone to intra-community variation [10–12]. It is therefore necessary to first describe these patterns of variation, in order to identify concentrations, or possibly even different types, of expertise in TEK. In situations where TEK is under threat of being lost (such as in a language endangerment scenario), an understanding of the usual patterns of knowledge transmission can help elucidate breakages in the transmission process [13, 14] and potentially help stop or reverse knowledge loss.

**Fig. 1:**
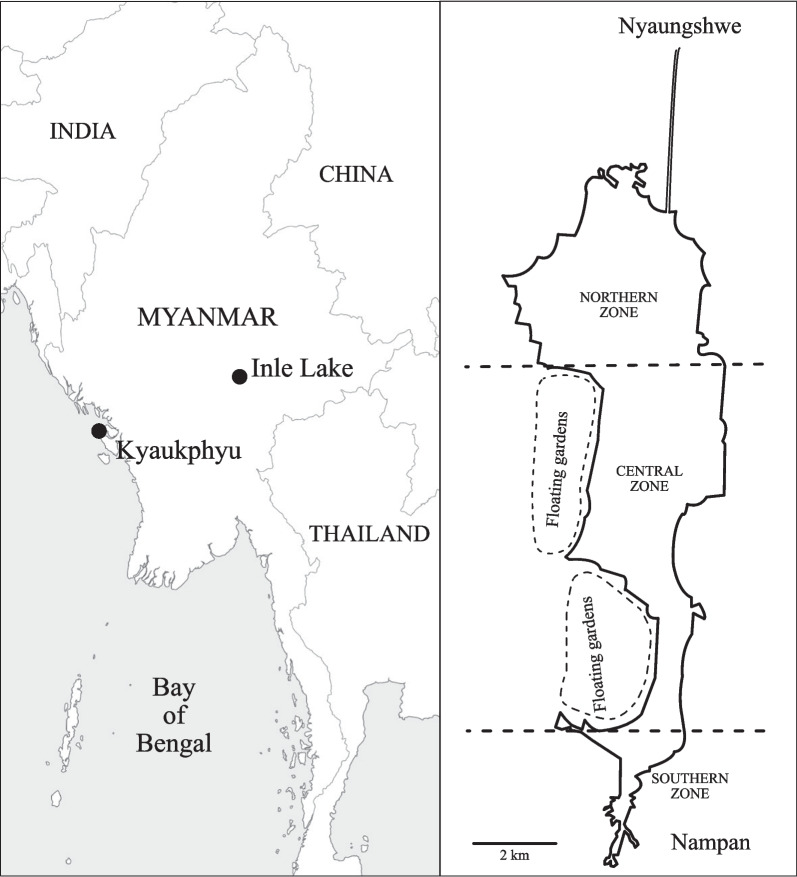
The locations of the field sites in Myanmar. The right panel shows Inle Lake in relation to the towns of Nyaungshwe and Nampan. The areas marked ‘floating gardens’ show parts of the lake with the highest concentrations of vegetable plots; in reality, such plots can be found all along the shore of the lake

The current study is an investigation of fish names in two very different fishing communities of Myanmar (Fig. 1). The first is Intha, who live on the shores of Inle Lake, located in southern Shan State, Myanmar. The Lake lies at an elevation of around 880 m and, in 2014, had an area of around 94 km^2^ [15]. With a speaker population of around 200,000 people spread over 64 villages, Intha can be considered a ‘stable’ language, albeit one that is closely related to the national language, Burmese. The main occupation among Intha men is fishing with gill nets and/or bamboo traps (the latter also being used for catching small shrimp), but some specialise in catching nearshore or stream fish with fishing spears. A significant number of villages, especially in the central region of the lake, have also taken up farming. This activity is carried out on large floating islands, and the main crop is tomato. There is also an established weaving industry in a handful of villages towards the south of the lake.

Inle Lake has been named a Ramsar wetland of international importance [16], but its ecosystem currently faces numerous challenges from tourism, agriculture, sedimentation and introduced species [17]. A common complaint among local fishermen is that their daily catches tend to be dominated by the introduced tilapia (*Oreochromis niloticus*, introduced about 20 years ago), while native species of great cultural importance are becoming harder to find (pers. obs., [18]). The changing ichthyofaunal composition of the Lake is reflected in the results of two species surveys carried out roughly a century apart. Possibly the first comprehensive inventory of fish species from Inle Lake is that of Annandale [19], who listed a total of 31 species, of which seven were newly described, and 12 were thought to be endemic. A more recent study by Kano et al. [20], which combined multi-sited sampling along with photographic documentation and DNA-barcoding, reported a total of 49 species, including 13 endemic species and 17 non-native species. Interestingly, Kano et al. mention that two of the endemic species mentioned in Annandale [19] were not found in their survey, and suggest that they might now be rare or extinct. It is against this backdrop of ecological degradation that the results of Intha TEK survey will be discussed.

The second language community investigated in this paper is the Rakhine language as spoken in and around the coastal town of Kyaukphyu on Ramree (pronounced [ɹə͂bɹɛ] locally; *ɹə͂*^*3*^* bɹɛ*^*3*^ with the tone notations used in Tables 5, 6) Island, Rakhine State. Rakhine also shares a large proportion of its vocabulary and grammar with standard Burmese, and with around one million speakers, can also be considered a ‘stable’ language. Rakhine artisanal fishermen typically fish close to the shore in small boats that hold one to two persons, or in larger boats of up to 30 feet and with a crew of 5 to 10 fishermen [21]. Drift gillnets and trammel nets are the most commonly employed technology, although baby trawls are also used sometimes [22]. Staked fence nets are also frequently used, and the resulting catch of small fish and shrimp is sorted and dried by women and children (pers. obs.) Often, a fishing trip starts early in the morning and lasts until the afternoon, but some fishermen leave in the evening and stay out at sea overnight. During the evening, couples can be seen catching small fish with beach seine nets in the intertidal zone, while night fishing (mostly for squid) is also carried out by many from piers and seawalls or from small vessels, using lamps as lures.

Working in the waters of the Bay of Bengal, Rakhine fishermen could potentially encounter hundreds of fish species; indeed, the ichthyological database Fishbase (www.fishbase.se) lists 615 marine species as occurring in Myanmar. It has been suggested that overfishing and habitat loss have led to a significant decrease in overall fish biomass in the last few decades. Longer lived, high value species (for instance, predatory species such as groupers, snappers and emperors), as well as sharks and rays, have been affected particularly badly [23].

Intha fish names do appear in Annandale [19], but some names appear to have changed, and the transcriptions are in general not very precise. A more recent publication [17] contains a more accurate name list, but for only a subset of the total species. Moreover, some of the scientific names used in that article are outdated. In the case of marine fish species, Psomadakis et al. [23] provide some Burmese local names, but it is unclear which, if any, are also used in the Rakhine language. As a result, there is an urgent need to compile an updated and comprehensive list of fish names for both communities.

The communities mentioned above are similar in that artisanal fishing is a primary activity among men in both locations. Women play a crucial supporting role by processing and selling fish, as is customary in many parts of Asia [24, 25]. Moreover, fish, along with rice, can be regarded as staple in both communities, as the former is the main source of protein and is consumed every day in one form or another: fresh, dried or fermented. Both languages have strong speaker bases, which could imply that TEK transmission occurs normally within each population. Given these potentially favourable conditions for TEK, the present study aims to elucidate the normal patterning of fish TEK in both communities, with respect to key variables that have been investigated by numerous researchers in other parts of the world. These variables are age (e.g. [26]), gender (e.g. [27]) and occupation (e.g. [28]). In the context of the present study, the latter variable encompasses the categories fishermen, fish sellers and ‘lay’ persons who have nothing to do with fishing or the fish trade, but merely consume fish on a regular basis.

A key difference between the Rakhine and Intha communities, apart from the presence or absence of saltwater, respectively, is the number of species that individual community members must learn. Inle Lake’s fish assemblage of under 50 species represents a modest challenge and is presumably far easier to become familiar with than the formidable Bay of Bengal assemblage of just over 600 species. It could be hypothesised that fish TEK might be equally distributed among Intha people with little variation caused by factors such as age, gender or profession. On the other hand, the distribution of fish TEK could well be patchier among Rakhine speakers, as, for example, it might take years of experience for a young fisherman to become familiar with all the fish that are regularly caught in local waters (even if only a subset of the 615 species is actually exploited). The current study therefore aimed to investigate the Intha and Rakhine communities to uncover potential variation in fish naming ability (as a proxy for general ethnoichthyological knowledge) within those communities, and to determine the reasons for these differences. TEK can be influenced by cultural, political, environmental or economic forces [29], but the current study focuses on biological and cultural factors, as these are more in line with the expertise of the authors.

In summary, the primary aims of this study were as follows:

1. Compile a list of local fish names in two fishing communities of Myanmar (Intha, lacustrine, and Rakhine, coastal marine).
2. Identify patterns of ethnoichthyological lexical knowledge in the two communities.
3. Discuss similarities and differences between the two communities to determine key mechanisms responsible for the observed patterns in knowledge.

## Methods

Field trips to Inle Lake and Kyaukphyu (Fig. 1) were carried out in the winter (dry season) months of December, January and February in 2021–2022 and 2022–2023.

### Intha

A stimulus set containing 43 printed colour pictures of fish sourced from various websites was compiled for the purpose of eliciting Intha fish names. The species list for the stimuli was obtained from Kano et al. [20], who provide a comprehensive inventory of native and introduced fish species that can be found in Inle Lake. The authors were based in the town of Nyaungshwe (spelled Yawnghwe in older publications), where the first few interviews were carried out with fishermen and fish sellers. The majority of the interviews were carried out on the lake itself; the authors would approach working fishermen by means of a boat, and, once consent was obtained, carry out elicitation sessions, while the two vessels (the fisherman’s boat and the authors’ boat) floated alongside each other. Elicitation sessions were recorded with a Zoom H4n audio recorder, and the consultant’s (*i.e.* native-speaker interviewee’s) responses were simultaneously noted down on pre-prepared data-sheets. Elicitation sessions were also carried out in various Intha villages; villages on both the eastern and western shores of the lake were visited, as well as all along its north–south axis (Fig. 1). Interviews with Intha were carried out in the Burmese language. Note that the words ‘interviewee’, ‘respondent’ and ‘consultant’ are used interchangeably for the remainder of the paper.

For the purpose of data collection and analysis, Inle Lake was divided into three zones, namely North, Central and South (Fig. 1). This was done primarily because although fishing is carried out in more or less all Intha villages, people living in the Central villages tend more towards agriculture and grow crops on large floating islands. Moreover, many people in the Southern villages are employed in a weaving industry that is well known throughout Myanmar. It was of interest to uncover potential differences in fish knowledge among people living in the three zones. In addition, all respondents were classified by occupation as either ‘current fishermen’, ‘previous fishermen’, people who had ‘never fished’ and ‘sellers’ (Table 1). The category of ‘previous fishermen’ comprised of (usually older) men who had fished full- or mostly-full-time in the past, but were now engaged in other activities (agriculture or tourism, for example). The ‘sellers’ were all women, who sold fish at local markets. The ‘never fished’ category consisted of men and women of all ages and included weavers, workers in the hospitality industry, cigar rollers and boatmen, among others. Note that all Intha young boys do go out fishing every now and then, either to catch a meal for their family or for leisure. The individuals in the following analyses who had ‘never fished’ had never done so as adults. The ‘never fished’ group included 11 females and 9 males.

**Table 1:** Number of Intha respondents in each Zone and Occupation category, and number of Rakhine respondents in each Occupation category

Occupation (Intha)	Current fishers	Previously fished	Never fished	Fish sellers	Total
*Zones*					
North	12	11	3	7	*33*
Central	7	15	6		*28*
South	9	4	11	4	*28*
Total	*28*	*30*	*20*	*11*	*89*

Occupation (Rakhine)	Fishermen		Consumers	Sellers (+ brokers)	Total
	39		24	24 (+ 3)	*90*

Note that Intha ‘never fished’ and Rakhine ‘consumers’ are analogous

Italics indicates highlighting the totals

Statistical analysis was carried out in the IBM SPSS 27 Statistics package, with the independent variables being respondent Age, Zone and Occupation. The dependent variable Total was the total number of fish stimuli (pictures) identified and named by a consultant. Imagine a scenario where one respondent identifies 15 fish pictures and provides 15 different names for those fish, while another respondent also identifies 15 pictures, but only provides 10 different names. In this situation, respondent 2 can be said to know fewer names than respondent 2; however, by using a handful of names more than once, she/he is able to identify the same Total number of species as respondent 1. The variable Total is therefore not very informative, as even a consultant who only knows a few fish names could use those names to label all the stimuli presented to him/her (*i.e.* by using the same name to label a number of fish species). The resulting dataset would presumably include several misidentifications. A more meaningful dependent variable, Unique, was the number of unique (non-repeated) fish names volunteered by each respondent. This variable gives a better estimate of the size of an individual’s ethnoichthyological lexicon. Kruskal–Wallis tests were carried out to investigate the effect of Zone and Occupation. With respect to Age, consultants from all Occupation and Zone categories were grouped into three Age categories (Table 2), namely ‘younger’ (up to 29 y.o.), ‘intermediate’ (30–49 y.o.) and ‘older’ (50 or more y.o.), and a Kruskal–Wallis test was carried out to determine whether there was a difference in fish knowledge among the three groups. A Mann–Whitney test was carried out to compare male and female responses for the ‘never fished’ group only.

**Table 2:** Number of Intha and Rakhine respondents in each Age category

Category	Intha count	Av. Age ± S.D	Rakhine count	Av. Age ± S.D
Younger	18	22.2 ± 4.49	19	24.0 ± 5.64
Intermediate	26	37.9 ± 5.22	46	41.6 ± 6.3
Older	45	56.8 ± 7.01	25	57.4 ± 8.57
*Total*	*89*		*90*	

Italics indicates highlighting the totals

### Rakhine

A list of species appropriate to the region was first compiled using the regional checklist function on the website Fishbase (www.fishbase.se). Although the complete checklist contained over 600 marine fish species found in Myanmar waters, a selection of 218 species was made, while ensuring that all families were represented. Species that were likely to be commercially important were given preference, but a number of smaller, probably inedible species were also included. A stimulus set of colour pictures was sourced from the Internet, and printouts were used to elicit Rakhine fish names. The vast majority of interviews were carried out in the coastal town of Kyaukphyu, where the authors were also based. Numerous fishing villages, home to artisanal fishermen, can be found on the outskirts of Kyaukphyu, and several large commercial fishing trawlers are also berthed at the local docks. Additionally, a number of interviews were carried out in the nearby coastal village of Kyaukpyauk, where many fishing families also live, as well as in the inland town of Rambrae (or Ramree; it shares its name with the island as a whole). Data from the latter location have not been used in this paper, as the locals fish in tidal creeks, and are exposed to a different set of fish species. Elicitation sessions were mostly carried out at the homes of the fishermen and fish sellers, or in the hotel where the authors were residing, but a small number took place during fishing trips. Interviews with Rakhine people were carried out in the Rakhine language.

Age-based analyses were carried out using the same categories as for the Intha data (Table 2). To investigate the effect of occupation, the authors had originally intended to collect data from three categories of respondents, namely ‘fishermen’, ‘fish sellers’ (who are almost always women) and ‘consumers’ (the latter referring to people in the general community, male and female, with no direct involvement in the fish trade). However, three of the ‘sellers’ interviewed turned out to be fish brokers (intermediaries who buy fish wholesale from fishermen and re-sell it to fish sellers); these were placed in the separate category ‘brokers’ during the analyses, due to their very different life experiences and interactions with fish (Table 1). All three were males, but it should be pointed out that there are several female brokers in Kyaukphyu. The ‘consumer’ group contained 11 females and 13 males.

As with the Intha data, statistical analysis was carried out in the IBM SPSS 27 Statistics package, with the independent variables being respondent Age and Occupation. (Brokers were excluded from the latter analysis due to the small sample size.) The dependent variable Total was the total number of fish identified and named by a consultant. The dependent variable, Unique, was the number of unique (non-repeated) fish names volunteered by each respondent. Similar to the Intha analysis, the variable Unique provides a better indication of a consultant’s knowledge of local fish ethnospecies. In addition, a third dependent variable, Core, was calculated to remove the large number of fish species, in the stimulus list, which did not have distinctive names, and/or were rare, commercially unimportant or avoided for being unpleasant or dangerous. The Core name list contained only 95 species (these are the fish for which respondent counts are provided in Table 4) and included fish that could be named confidently by expert fishermen, were commercially and/or culturally important and were not labelled solely by broad category labels, such as ‘pufferfish’, ‘stingray’ or ‘flatfish’. Some highly recognisable fish species, such as mudskippers and flying fish, were also excluded from the Core list, as they provided no challenge to respondents. As a result, the Core list came to include fish species that were relevant to the local culture, and which nevertheless required some level of expertise to identify correctly. Respondents were said to have named a Core fish correctly only if the name provided was in broad agreement with the name(s) used by expert fishermen for that species. Kruskal–Wallis tests were carried out to investigate the effect of Occupation on Unique and Core, while Kendall’s Tau was calculated to determine the presence of a correlation between Age and Unique. As the ‘consumers’ category of respondents contained both males and females (54% males, 46% females), a Mann–Whitney U test was carried out to determine if gender had an effect on Unique or Core.

## Results

### Name inventory: Intha

Table 3 shows the Intha names recorded for the Inle Lake fish species, along with the number of respondents who were able to correctly identify each species. Of the 43 stimulus species used for elicitation with Intha respondents, four (*Chaudhuria caudata*, *Glyptothorax rugimentum, G. siamensis* and *Oryzias uwai*) could not be identified by anyone, while a handful of other species (such as the endemic *Poropuntius schanicus*) were only recognised by a small minority (less than ten) of respondents. These were mostly smaller species, with a size range of around 10–13 cm. There were, however, a number of smaller species that many people recognised: these included the tank goby *Glossogobius giuris*, the loaches *Lepidocephalichthys berdmorei* and *Petruichthys brevis*, the cyprinids *Inlecypris auropurpureus*, *Pethia stoliczkana* and *Puntius sophore* and the glassfishes *Parambassis* spp. A number of these were reported as forming an important part of the locally consumed ‘dried whitebait’ by Annandale [19] (it is known locally as *ngət̪əphwɛ*^*3*^* chauʔ*), and this tradition continues to this day. An important difference between Annandale’s observations and the present study lies in the abundance of the small endemic species *Sawbwa resplendens*, with a maximum length of 3.5 cm. Annandale reports it as being ‘extremely abundant all over Inle Lake’ and ‘of economic importance’ (p. 49), but only 18 older individuals were able to identify it in the present study.

**Table 3:** Intha names recorded for local fish species from numerous villages around Inle Lake

# respondents	Scientific name	Intha name(s)
21	*Anabas testudineus*	nga^3^ thauʔ t̪wa^3^, ngəbye^2^ ma^1^
20	*Balitora* sp.	nga^3^ tein^2^ kaʔ, nga^3^ tein^2^ tɛʔ
74	*Barbonymus gonionotus*	nga^3^ phyu^2^, thain^3^ [< Thai] ngəkhoun^3^ ma^1^, jəpan^2^ [< Japan] ngəkhoun^3^ ma^1^, nga^3^ khoun^3^ ma^1^, zaw^2^ ji^2^ ngəkhoun^3^ ma^1^, ye^2^ cho^2^ nga^3^ khoun^3^ ma^1^
9	*Celestichthys erythromicron*	(ngət̪əphwɛ^3^), (ngəcɛ^2^ pyauʔ)
77	*Channa harcourtbutleri*	nga^3^ u^2^ mɛ^1^
71	*Channa striata*	nga^3^ yan^1^
0	*Chaudhuria caudata*	Unknown
77	*Clarias* cf.* batrachus*	nga^3^ khu^2^
83	*Clarias gariepinus*	nga^3^ khu^2^, a^2^ fəri^3^ ka^3^ [< Africa] ngəkhu^2^, ət̪a^3^ sa^3^ ngəkhu^2^
46	*Ctenopharyngodon idella*	myɛʔ sa^3^ nga^3^, myɛʔ sa^3^ ngəjin^3^, hmɔ^1^ sa^3^ nga^3^, hmɔ^1^ sa^3^ dɛ^1^ ngəjin^3^
86	*Cyprinus intha*	nga^3^ phein^3^
40	*Cyprinus rubrofuscus*	nga^3^ shwe^2^ wa^2^, mwe^3^ ngəphein^3^, shwe^2^ wa^2^ nga^3^ phein^3^
8	*Devario kakhienensis*	(pyɛʔ sa^3^ yu^3^ ma^1^)
3	*Esomus danrica*	nga^3^ ye^2^ bɔ^2^
17	*Gambusia affinis*	nga^3^ baiʔ phaun^3^, nga^3^ baiʔ ka^3^, nga^3^ u^3^ phaun^3^
15	*Garra gravelyi*	nga^3^ loun^3^, poss. also the names used for *Balitora* sp.
71	*Glossogobius* cf.* giuris*	nga^3^ khaun^3^ pwa^1^, ngəloun^3^ jan^3^
0	*Glyptothorax rugimentum, G. siamensis*	Unknown
85	*Gymnostomus horai*	nga^3^ lu^3^, ngəjin^3^ phyu
85	*Heteropneustes fossilis*	nga^3^ ci^3^, ngəkhu^2^ yo^3^ yo^3^, ngəji^3^
65	*Inlecypris auropurpureus*	nga^3^ hnyaun^2^ she^2^, nga^3^ ni^2^ sein^3^, ngəye^2^ bɔ^2^
24	*Labeo rohita*	ngəmyiʔ chin^3^, nga^3^ myɛʔ hsan^2^ ni^2^, ngəjin^3^ myɛʔ hsan^2^ ni^2^
52	*Lepidocephalichthys berdmorei*	nga^3^ səlɛ^3^ tho^3^
76	*Mastacembelus caudiocellatus*	nga^3^ hmwe^2^, ngəmwe^2^ tho^3^
34	*Microrasbora rubescens*	ngət̪əphwɛ^3^, nga^3^ t̪əphwɛ^3^ chauʔ
87	*Monopterus cuchia*	ngəshɛn^1^, shin^1^ pɛn^3^
87	*Monopterus javanensis*	ngəshɛn^1^, shin^1^ phyu^2^
86	*Notopterus notopterus*	nga^3^ phɛ^2^, ngəphɛ^2^ ma^1^
86	*Oreochromis niloticus*	cauʔ nga^3^, si^2^ la^3^ pi^3^ ya^3^, nga^3^ wain^3^
0	*Oryzias uwai*	Unknown
81	*Parambassis lala, P. ranga*	pəlaʔ səteiʔ [< plastic] nga^3^, nein^2^ lun^2^ [< nylon] nga^3^, ngəhman^2^, hman^2^ nga^3^
58	*Pethia stoliczkana*	nga^3^ hmɛ^1^ tiʔ, nga^3^ hmɛ^1^, ngəmɛ^1^ dəbauʔ, nga^3^ təlɛʔ ma^1^
32	*Petruichthys brevis*	nga^3^ ni^2^ pyauʔ, ngəpyauʔ, ngəyiʔ plauʔ, nga^3^ pyauʔ ma^1^
22	*Physoschistura shanensis*	ngət̪əlɛ^3^ tho^3^, (nga^3^ phɛ^2^ souʔ ma^1^)
15	*Poecilia reticulata*	daun^3^ nga^3^
5	*Poropuntius schanicus*	nga^3^ yiʔ
53	*Puntius sophore*	zɔ^2^ gyi ngəkhoun^3^ ma^1^, ngəkhoun^3^ ma^1^ hmɛ^1^ dəbauʔ, ye^2^ cho^2^ nga^3^ khoun^3^ ma^1^
18	*Sawbwa resplendens*	nga^3^ bəzaʔ ni^2^, nga^3^ mi^3^ ni^2^, mi^3^ ni^2^ gaun^3^ ni^2^, nga^3^ saw^2^ bwa^3^
70	*Systomus* cf.* rubripinnis*	ngəkhoun^3^ ma^1^ mi^3^ ni^2^, nga^3^ khoun^3^ ma^1^, de^2^ t̪a^1^ ngəkhoun^3^ ma^1^, in^3^ d̪a^3^ ngəkhoun^3^ ma^1^
27	*Trichogaster labiosa*	nga^3^ mɛ^3^, nga^3^ pouʔ pya^3^, nga^3^ pya^2^
40	*Trichopodus pectoralis*	ngəwɛʔ, nga^3^ wɛʔ ma^1^

Column 1 gives the number of people (from a total of 89 respondents) who provided at least one of the names listed in Column 3. Scientific names are the updated versions and may differ from those given in Annandale [19]. See Fuke et al. [30] for name correspondences

*N.B.* The first name in each cell of Column 3 is the most commonly recorded Intha name. Names in parentheses indicate ethnotaxon labels that were recorded infrequently; they may be from a different dialect or, in rare cases, may represent an incorrect identification. < followed by an English word indicates a loanword in the name. One species from the Kano et al. [20] survey, *Neolissochilus nigrovittatus*, was not included, as no suitable photograph could be found

*Pronunciation note* A modified (simplified) version of the International Phonetic Alphabet has been used here to maximise precision and intelligibility to a wider audience: *ə*, schwa, as in the ‘a’ of ‘about’; *ɔ*, as in the ‘au’ of ‘caught’; ɛ, as in the ‘e’ of ‘get; *ʔ*, glottal stop; *t̪* and *d̪* indicate dental consonants; *hs*, ‘hissy s’ [s^h^]; *hl*, *hm*, *hn* and *hr* are devoiced *l* [l̥], *m* [m̥], *n* [n̥] and *r* [r̥], respectively; *ph*, *th* and *kh* represent the aspirated stops [p^h^], [t^h^] and [k^h^], respectively; *sh* and *ch* are pronounced as in English; *ng*, velar nasal [ŋ]; *c* is the affricate [tɕ]; *n* at the end of a syllable (*e.g. oun*) indicates a nasal vowel; and superscripts ^1^ ‘creaky’ tone, ^2^ ‘low’ tone, ^3^ ‘high’ tone

Another endemic species, *Physoschistura shanensis*, sampled by Kano et al. [20] from streams to the west of Inle Lake proved not to be very well known, with only 22 respondents giving it a name. The small *Microrasbora rubescens* fared slightly better, as it was identified by 34 respondents. Even this higher number is at odds with Annandale’s observation that ‘this little fish is very abundant all over Inle Lake’ (p. 51), and that it also forms an important constituent of dried whitebait.

Many larger fish species are widely known and represent ethnotaxa that are presumably still common and/or culturally important. These include the *nga*^*3*^* phein*^*3*^ (*Cyprinus intha*), the most highly prized of all fish to Intha people, and almost universally known, along with other large carp (some of which are native, such as *Systomus rubripinnis,* while others are introduced aquaculture species, such as the Java/silver barb *Barbonymus gonionotus*), catfishes (*Clarias* spp.), eels (*Monopterus* spp.), snakeheads (*Channa* spp.) and the bronze featherback (*Notopterus notopterus*). Not surprisingly, the highly invasive and fecund Nile tilapia *Oreochromis niloticus* is also universally recognised. This fish probably dominates the Inle Lake ecosystem now, as nearly all fishing boats encountered by the authors during data collection (except for those focusing on shrimp) contained nothing but tilapia.

### Name inventory: Rakhine

The Rakhine fish names recorded in Kyaukphyu and Kyaukpyauk are presented in Table 4, along with the number of respondents who were able to correctly identify the species in the Core list. While many ‘local’ names are presented in Psomadakis et al. [23], there is no information on where the names were collected, and it is likely that very few are in the Rakhine language. Moreover, many of the names are direct translations of the official English Food and Agricultural Organisation (FAO) names into Burmese, as in ‘Taung Pan Mae Set Nga Pyan’ for ‘spotfin flyingfish’ (p. 341). Thus, while Psomadakis et al*.* is resource of outstanding value, the local names presented within this publication cannot be considered as a substitute for primary language documentation in fishing communities.

**Table 4:** Rakhine names recorded in Kyaukphyu and Kyaukpyauk for various marine fish species

# responses	Scientific name(s)	Rakhine name(s)
15	*Abalistes stellatus*	t̪an^2^ mənain^2^ cauʔ mənain, ngət̪an^2^, bəjin^3^ əkhun^2^ thu^2^
	*Ablabys macracanthus*	ngəʔouʔ, cauʔ bəlu^3^, nga^3^ bəlu^3^
88	*Ablennes hians*	ngətaun^1^ hnyin^3^, ngəphaun^2^ ro^3^, ngətho^3^ dan^2^, (ngəsəloun^3^)
	*Abudefduf septemfasciatus*	cauʔ ngəbri^2^ ma^1^
82	*Acanthopagrus berda*	ngəwɛʔ
	*Acanthurus leucosternon*	ngəran^2^ hra^2^ ma^1^, ngəhla^1^, cauʔ nga^3^
	*Acanthurus tennentii*	cauʔ ngəran^2^ hra^2^ ma^1^
	*Acentronura breviperula*	ri^2^ nəga^3^
	*Aetobatus narinari*	swan^2^ krwi^2^, in^3^ bauʔ
	*Aetomylaeus nichofii*	leiʔ cauʔ swan, əchɔ^3^, di^3^ douʔ
40	*Albula oligolepis*	ngələwa^2^, ngətein^3^, ngət̪e^3^ tho^3^
47	*Alectis indica*	bya^2^ da^2^ waiʔ, youʔ so^3^ ma^1^, ngəwain^3^ she^2^, ngənan^2^ ba^3^
	*Alepes djedaba*	mi^3^ wa^2^, bədoun^3^ wa^2^
	*Allenbatrachus grunniens*	nga^3^ bəlu^3^, ngəʔouʔ
15	*Aluterus monoceros*	nga^3^ sɛʔ ku^2^, seʔ ku^2^ bəjin^3^
	*Ambassis gymnocephalus*	kraun^2^ məsa^3^
	*Amblyeleotris latifasciata*	ngətauʔ te^1^, ngətaun2 tu^2^
71	*Amblygaster leiogaster*	ngəkoun^3^ nyo^2^, ngəcɔ^3^ nyo^2^ (pya^3^)
63	*Amblygaster sirm*	ngəcɔ^3^ nyo^2^ (loun^3^)
46	*Anguilla bengalensis*	ngəpəthoun^3^, ngəphəroun^2^, ngəshan^3^ ga^3^, (ngəyoun^2^)
	*Anguilla bicolor*	ngəhauʔ, wa^2^ ji^3^ ma^1^ əri^2^, (ngəʔouʔ), (ngəshan^3^ ga^3^)
90	*Anodontostoma chacunda*	ngəwan^3^ bu^2^
	*Anoxypristis cuspidata*	swe^2^ t̪i^2^, rain^2^ t̪i^2^, ngəman^3^ swe^2^ t̪i^2^
19	*Antigonia emanuela*	nga^3^ t̪an^2^ ze^2^
	*Aprion virescens*	nət̪i^3^ shwan^3^, cauʔ ngəshwan^3^, pein^2^ shwan^3^
17	*Argyrops spinifer*	ngəni^2^ [ma^1^], ngəwɛʔ [saʔ/ phyu], (a^2^ khaun^2^ ni^2^), (shain^2^ baun^2^ d̪i^3^)
87	*Arius arius*	ngəhsu^2^, ngəran^3^ goun^3^, ngəzərain^3^
76	*Arius maculatus*	ngəran^3^ goun^3^
	*Atelomycterus marmoratus*	ngəman^3^ tauʔ te^1^
70	*Atrobucca nibe*	ngəbouʔ tin^2^, hsaʔ pha^2^
	*Atropus atropos*	bya^2^ zan^2^ waiʔ, ngəwain^3^
37	*Atule mate*	pəla^2^ tu^3^ [mi^3^ wa^2^/yauʔ pha^1^/yauʔ ma^1^], (ngəmo^3^ d̪i^3^)
	*Aurigequula fasciata*	ngəsəne^3^, ngəzi^3^ ne^3^, ngədan^3^ ga^3^
48	*Auxis rochei*	ngəme^3^ loun^3^, ngəʔoun^3^ d̪i^3^, nga^3^ pəlɔn^3^, (ngəpouʔ yaun^2^), (nga^3^ ci^3^ gan^3^)
43	*Auxis thazard*	nga^3^ oun^3^ d̪i^3^, ngəmɛ^3^ loun^3^, nga^3^ pəlin^3^, (ngəpouʔ yaun^2^), (nga^3^ ci^3^ gan^3^)
5	*Bahaba chaptis*	(ngəbaun^3^ zauʔ), (bədoun^3^ she^2^), (mi^3^ dan^2^ she^2^), (ngəphauʔ lauʔ)
5	*Bathygobius meggitti*	ngətoun^3^ pwa^1^, ngət̪əpwa^1^
	*Batrachocephalus mino*	ngəshɔ^3^ shəphya^2^, ngəhsu^2^, ngəran^3^ goun^3^ cauʔ phya^2^
	*Bodianus neilli*	nət̪i^3^ shwan^3^ (cauʔ nga^3^), cauʔ ngəshwan^3^
87	*Boleophthalmus boddarti*	ngəyɛʔ pyauʔ, ngətaun^2^ tu^2^, ngədaun^3^ byauʔ
	*Canthigaster cyanospilota*	bəjɔn^3^
	*Canthigaster petersii*	bəjɔn^3^
	*Carangoides armatus*	bya^2^ da^2^ waiʔ, ngənan^2^ bya^3^, ngəwain^3^, bya^2^ dan^2^ waiʔ, ngənan^2^ ba^3^, bya^2^ bya^2^ waiʔ
44	*Chanos chanos*	ngətein^3^, mraiʔ ngələwa^2^, chaun^3^ nga^3^
	*Cheilinus undulatus*	cauʔ ngətauʔ tu^2^, cauʔ ngəji^3^
	*Cheilopogon abei*	ngəbyan^2^
	*Chelonodon patoca*	bəjɔn^3^, ngəpu^1^ tin^3^
	*Chiloscyllium burmense*	ngəman^3^ tauʔ te^1^
71	*Chirocentrus nudus*	ngədəhrwe^2^, ngədəlwɛ^2^
	*Chlorurus troschelii*	ngəci^3^
68	*Coilia dussumieri*	ngəpaun^3^ sauʔ, bədoun^3^ she^2^, mi^3^ dan^2^ she^2^, (ngəphauʔ lauʔ)
56	*Coryphaena equiselis, C. hippurus*	ngədəmauʔ, ngəkhaun^3^ ba^3^ [yauʔ pha^1^]
80	*Crenimugil seheli (Mugil cephalus)*	ngəkan^2^ jin^3^ [byan^2^], bədoun^3^ nyi^3^, gaun^3^ jan^3^, (ngənyein^2^), (ywɛʔ ywin^3^)
	*Cynoglossus bilineatus*	ngəsha^2^, ngəphɛʔ ywɛʔ
	*Cynoglossus puncticeps*	ngəsha^2^, ngəphɛʔ ywɛʔ, ngəsha^2^ prauʔ, ngəsha^2^ ca^3^
41	*Decapterus russelli*	pəla^2^ tu^3^
	*Dendrophysa russelii*	ngəbouʔ t̪ɔn
	*Deveximentum insidiator*	ngədan^3^ ga^3^
	*Diagramma pictum*	ngət̪əkhauʔ, ngət̪əkhauʔ prauʔ ma^1^, po^3^ da^2^ li^2^ t̪ein^3^ dan^2^, cauʔ ngəraiʔ ma^1^, nəkhan^3^ lan^2^, cauʔ nga^3^ shwe^2^ lɛ^2^ t̪u^2^
	*Diodon hystrix*	bəjɔn^3^ kət̪aiʔ, su^3^ soun^2^ bəjin^3^, ngəlaun^2^ lin^2^
75	*Drepane punctata*	hsin^2^ nərwɛʔ, ngəhsin^2^ na^3^, əhrin^2^ bouʔ
	*Dussumieria acuta*	ngəkrɔ^3^ nyo^2^ əloun^3^, ngəmo^3^ d̪i^3^, ke^2^ se^2^ ye^3^ nga^3^, byan^3^ byan^3^ kwe^3^
54	*Echeneis naucrates*	nga^2^ phənaʔ, ngəgaʔ
8	*Echidna nebulosa*	əʔo^2^ ma^1^ əhri^2^, ngəji^3^ ma^1^ əhri^2^, ngəshan^3^ ga^3^, wa^2^ ji^3^ ma^1^ hri^2^
	*Elagatis bipinnulata*	ngətein^3^, ngəshwan^3^, cɔ^2^ hein^3^
88	*Eleutheronema tetradactylum*	ngətəya^3^
48	*Elops hawaiensis*	ngətein^3^ [loun^3^], [mraiʔ] ngələwa^2^
10	*Epinephelus* spp.	ngətauʔ tu2
60	*Equulites elongatus*	ngədan^3^ ga^3^ oun^3^ baun^2^
	*Equulites leuciscus*	ca^3^ ngədan^3^ ga^3^, ngədan^3^ ga^3^
79	*Escualosa thoracata*	saiʔ səli^2^, ngəhla^1^ she^2^, bədi^3^ phyu^2^, ngət̪in^3^ thoun^3^, ngəphyu^2^ [loun^3^/ she^2^], (ngəzan^2^ phyu^2^), (maun^2^ pyan^2^ ke^3^), (bədan^3^ t̪əma^1^)
	*Eubleekeria splendens*	ngədan^3^ ga^3^ [su^3^ ma^2^]
90	*Eupleurogrammus muticus*	ngət̪əhrwe^3^, maun^3^ ja^3^
49	*Eusphyra blochii*	ngəman^3^ cwe^3^
49	*Euthynnus affinis*	ngəʔoun^3^ d̪i^3^, ngəpouʔ yaun^2^, ngəmɛ^3^ loun^3^, nga^3^ pəlin^3^, ngəci^3^ gan^3^
	*Exocoetus volitans*	ngəbyan^2^, ngətein^3^ byan^2^
60	*Filimanus xanthonema*	ngəlɛʔ khwa^2^ bauʔ, ngətəya^3^, ngəcaun^3^ dəbe^1^, ngəpoun^2^ na^3^
41	*Fistularia petimba*	ngəphaun^2^ ni^2^, hsəleiʔ pain^3^ sa^3^ ma^1^, ngəlein^2^, (ngəʔaʔ)
	*Galeocerdo cuvier*	ca^3^ ngəman^3^, ngəman^3^ gaun^3^ waiʔ, ngəman^3^ ja^3^,, ngəman^3^ t̪e^3^ tho^3^, ngəman^3^ gaun^3^ touʔ
49	*Gempylus serpens*	ngətaun^2^ hnyin^3^ swe^2^ bya^3^, ngəphaun^2^ ro^3^ [cɔ^3^ sein^3^]
71	*Gerres erythrourus, G. oyena*	ngəhsi^2^ o^3^ [ma^1^], ngəsin^3^ zaʔ, (ngəzan^2^ phyu^2^)
13	*Glaucostegus granulatus*	ngəman^3^ kha^3^, ngəman^3^ pha^3^
	*Glossogobius giuris*	ngət̪əbraʔ t̪a^3^, ngətoun^3^ braʔ t̪a^3^, ngətoun^3^ pwa^1^
	*Glyphis siamensis*	ngəman^3^ gaun^3^ touʔ, ngəman^3^ t̪e^3^ tho^3^
55	*Gnathanodon speciosus*	ngəgouʔ [wa^2^], ngəʔoun^3^, (ngəkoun^3^)
	*Grammatobothus polyophthalmus*	ngəphɛʔ rwɛʔ, ngəlɛʔ kaiʔ, t̪e^3^ tho^3^ ma^1^
55	*Grammatorcynus bilineatus*	ngənyo^1^ pya^3^, (ngəchwan^3^)
	*Gymnosarda unicolor*	ngənyo^1^ loun^3^, (ngəchwan^3^)
	*Gymnothorax undulatus*	ngəphəyoun^2^, wa^2^ ji^3^ ma^1^ əri^2^
	*Halophryne diemensis*	nga^3^ bəlu^3^, ngəʔouʔ, (ngəthəyan^2^)
10	*Hapalogenys merguiensis*	cauʔ ngəwɛʔ, ngəwɛʔ mɛ^3^, nəkhwan^3^ byan^2^ ma^1^
84	*Harpadon nehereus*	ngədəmu^2^, ngəpyɔ^3^ d̪i^3^, bərəga^3^, ngəhnaʔ, a^2^ bre^3^
	*Hexanematichthys sagor*	ngərin^3^ goun^3^ cauʔ phya^2^, ngəran^3^ goun^3^ ngəmouʔ, ngəran^3^ goun^3^ theiʔ ki^3^, cauʔ phya^2^, ngətan^2^
71	*Hilsa kelee*	ngət̪əlauʔ, ngəmɔ^2^ tɔ^2^
	*Himantura uarnak*	leiʔ cauʔ krwi^2^
71	*Hyporhamphus limbatus*	ngətaun^2^ hnyin^3^, ngəphaun^2^ ro^3^, səleiʔ pain^3^ kaiʔ, səleiʔ pain^3^ sa^3^ ma^1^, ngəboun^3^ di^2^, daun^3^ di kaun^2^
57	*Ilisha megaloptera*	myɛʔ loun^3^ ce^2^, myɛʔ hsan^2^ ce^2^, myɛʔ loun^3^ pyu^3^ ma^1^, je^3^ byu^3^, (shauʔ pət̪ein^2^), (ngəba^3^)
18	*Iniistius bimaculatus*	ngəji^3^ [phyu^2^]
	*Istiompax indica*	ngəbran^2^ (nwɛ^3^) zaun^2^ rɔ^2^, laun^3^ tho^3^ nga^3^, nga^3^ zin^2^ yɔ^2^
74	*Johnius amblycephalus*	ngəbouʔ t̪ɔn^2^, hsaʔ pha^2^
69	*Katsuwonus pelamis*	ngəmɛ^3^ loun^3^, ngəcaun^2^, ngəzin^2^ yɔ^2^, nga^3^ pəlin^3^, ngəʔoun^3^ d̪i^3^, ngəbouʔ yaun^2^, phəyɛ^3^ d̪i^3^, (ja^2^ hsi^2^), (ngəmɔ^2^ youn^2^)
45	*Lactarius lactarius*	taun^2^ d̪a^3^
	*Lagocephalus inermis*	bəjaun^3^
94	*Lates calcarifer*	ngət̪ədaiʔ
60	*Leptomelanosoma indicum*	ngəlɛʔ khwa^2^
	*Lepturacanthus savala*	ngət̪əhrwe^3^ maun^3^ ja^3^
	*Lethrinus lentjan*	nət̪i^3^ chwan^3^, cauʔ ngəshwan^3^ ma^1^
	*Lutjanus malabaricus*	ngəni^2^ ma^1^
57	*Malacocephalus laevis*	mi^3^ dan^2^ she^2^, mi^3^ dan^2^ t̪we^2^, bədoun^3^ she^2^, ngəbaun^3^ zauʔ
76	*Megalaspis cordyla*	da^3^ mənain^2^, (ngədəpi^3^)
	*Megalops cyprinoides*	ngələwa^2^, shaun^3^ ngələwa^2^
	*Mene maculata*	tərouʔ da^3^
	*Mobula eregoodootenkee*	leiʔ cauʔ gwa^1^, ləcauʔ swɛn^2^ gwa^1^, leiʔ cauʔ cwe^3^
	*Monodactylus argenteus*	nga^3^ t̪an^2^ ze^2^
90	*Muraenesox cinereus*	t̪in^3^ baun^3^ tho^3^
	*Myripristis murdjan*	əkhwan^2^ thu^2^, əkri^3^ thu^2^ ma^1^, cauʔ əkri^3^ t̪oun^3^ thaʔ
28	*Narcine timlei*	nga^3^ daʔ laiʔ, dɔ^2^ ji^3^ yaun^3^ ma^1^, (nga^3^ lɛʔ thoun^2^)
	*Nematalosa nasus*	ngəwan^3^ bu^2^
60	*Netuma thalassina*	ngədan^2^, ngəran^3^ goun^3^, ngəhsu^2^, (chi^3^ thwɛʔ)
81	*Otolithes ruber*	ngəbouʔ t̪ɔn^2^ [ro^3^ hre^2^], hsaʔ pha^2^, ngəbouʔ t̪ɔn^2^ t̪wa^3^ t̪oun^3^ chaun^3^, ngəkraun^2^ ze^2^, ngəkraun^2^ swɛ^2^, (t̪in^2^ phyu^2^), (ngəprɛʔ loun^3^)
69	*Otolithoides biauritus*	[hsaʔ pha^2^] ro^3^ hre^2^, ngəbouʔ t̪ɔn^2^ [ro^3^ hre^2^], (ngəbouʔ t̪ɔn^2^ t̪əkrɛ^3^)
87	*Pampus argenteus*	yu^1^ zəna^1^, paʔ ta^2^ she^2^
58	*Pennahia anea*	ngəbouʔ t̪ɔn^2^ pu^1^, ba^2^ chɔ^3^, ngəbu^3^ ze^2^, ngəgaun^3^ ji^3^, ngəpyɛʔ kouʔ, ngəbouʔ t̪ɔn [pu^1^/ gaun^3^ wain^3^/ a^2^ gaʔ], (sha^2^ thouʔ ke^2^)
	*Pinjalo pinjalo*	ngəni^2^ ma^1^, ngəni she, ngəhla^1^ she^2^
90	*Planiliza macrolepis*	ngəkan^2^ jin^3^ (byan^2^), gaun^3^ jan^3^, ngəkan^2^ [jin^3^] d̪a^3^, chaun^3^ nga^3^, bədoun^3^ nyi^3^, (ngənyi^2^ nan^2^)
88	*Platycephalus indicus*	cwe^3^ bədoun^3^, ngəcwe^3^, ngəzin^2^ leiʔ, (ngəpraun^3^ khaʔ)
89	*Plotosus canius*	ngəkhu^2^
66	*Plotosus lineatus*	ngəke^3^, ngəkhəle^3^, ngəkre^3^
36	*Polydactylus sextarius*	ngəcaun^3^ dəbe^1^, ngətəya^3^, ngəlɛʔ khwa^2^, ngəpoun^2^ na^3^, (auʔ mouʔ seiʔ), (hmyain^2^ kho^3^)
	*Pomadasys olivaceus*	ngəkhəyu^1^
31	*Pristis microdon*	ngəman^3^ hswe^2^ t̪e^2^, ngəman^3^ hlwa^1^, ngəman^3^ swe^2^ zoun^2^, ngəman^3^ rain^3^ t̪e^2^, hlwa^1^ ngəman^3^
83	*Protonibea diacanthus*	ngəbouʔ t̪ɔn əmɛ^3^, hsaʔ pha^2^, (ngəpyɛʔ loun^3^ əkhɛ^3^)
46	*Pterotolithus maculatus*	əphyain^3^ za^3^, ngəbouʔ ca^3^, t̪an^2^ wa^2^, ngəbouʔ t̪in^2^ pyauʔ ma^1^
17	*Raconda russeliana*	ngədəla^2^, (ngəmyɛʔ khɛ^3^)
79	*Sardinella fimbriata, S. gibbosa*	ngəkoun^3^ nyo^2^, ngəcɔ^3^ nyo^2^
19	*Sargocentron praslin*	əkri^3^ thu^2^, ngəci^3^ ma^2^, ngəran^2^ hra^2^ ma^1^, (ngəʔi^3^), (ngəni^2^ kri^3^ jan^3^), (əkhun^2^ thu^2^ [ngəni^2^])
30	*Saurida tumbil*	ngəzin^2^ leiʔ, mraiʔ ngəsəloun^3^, kəla^3^ li^3^, (ngəpəlwe^2^)
88	*Scatophagus argus*	ngəpət̪oun^2^, bi^2^ chaʔ təya^2^
80	*Scomberoides commersonnianus*	ngəkhin^3^ ba^3^
	*Scomberoides tol*	ngəkhin^3^ ba^3^ əkhwan^2^ thu^2^, ngəkhin^3^ ba^3^ əri^2^ thu
	*Scomberomorus guttatus*	ngəshwan^3^, ngənyo^1^ loun^3^, (bi^3^ zin^3^ loun^3^)
24	*Selar crumenophthalmus*	pəla^2^ tu^3^ yauʔ pha^1^, pəla^2^ tu^3^ myɛʔ pyu^3^, myɛʔ loun^3^ ce^2^
42	*Selaroides leptolepis*	mi^3^ wa^2^ she^2^
49	*Setipinna tenuifilis*	ngəba^3^ (she^2^), ngəba^3^ ma^1^, a^2^ bre^3^, ngəpəsha^3^
76	*Siganus canaliculatus*	t̪oun^3^ phya^3^ i^3^, ngəran^2^ hra^2^ ma^1^, (ngəshəya^3^ ma^1^)
74	*Sillaginopsis panijus*	pin^2^ lɛ^2^ ngəsəloun^3^, nga^3^ khran^2^ dain^2^, ngəzan^2^ bu^2^, (t̪aun^3^ phɔ^3^ phauʔ), (t̪e^3^ tho^3^)
	*Sillago sihama*	ngəzwan^2^ bu^2^, t̪e^3^ tho^3^ su^3^, t̪e^3^ tho^3^ nga^3^
73	*Sphyraena jello*	ngəlwan^3^, ngədəhsauʔ, (ngədouʔ)
74	*Sphyraena obtusata*	ngəlwan^3^, ngədəhsauʔ, (ngədouʔ)
79	*Stolephorus commersonnii, S. indicus*	ngəni^2^ tu^2^, ngəloun^3^ she^2^, (wɛʔ touʔ)
83	*Strongylura leiura*	ngətaun^2^ nyin^3^, ngəphaun^2^ ro^3^, hsəleiʔ pain^3^ sa^3^ ma^1^, (cɔ^3^ zein^3^ chun^2^)
	*Taeniura lymma*	leiʔ cauʔ twan^3^ khan^2^, leiʔ cauʔ phon^3^ gri^3^, leiʔ cauʔ sin^3^, tin^3^ khwɛ^3^ leiʔ cauʔ, leiʔ cauʔ san^3^ pyan^3^
76	*Tenualosa ilisha*	ngət̪əlauʔ, ngəmɔ^2^ tɔ^2^
70	*Terapon jarbua*	ye^2^ nan^2^ kraun^2^, ngənan^2^ kraun^2^, (ngənan^2^ kraun^2^ t̪əkho^3^), (hnɛ^3^ hsəya^2^)
50	*Thryssa hamiltonii*	myɛʔ loun^3^ ce^2^, myɛʔ hsan^2^ ce^2^, a^2^ bye^3^, a^2^ je^2^ ma^1^, ngəba^3^ she^2^, ngəpəsha^3^
	*Thryssa mystax*	ngəpəsha^3^, ngəba^3^ she^2^, a^2^ bye^3^
	*Trachinotus baillonii*	ngəkhaun^3^ ba^3^
41	*Triacanthus nieuhofii*	nga^3^ phɔ^2^ ka^2^ [< Fokker], hɛ^2^ li^2^ kɔ^2^ pəta^2^ [< helicopter], haʔ səki^3^ [Husky], gwa^1^ dauʔ, jo^2^ du^1^, ngəhsin^2^, le^2^ yin^2^ byan^2^, jɛʔ [< jet] le byan, (ngəmyin^3^)
42	*Trichiurus lepturus*	ngət̪əhrwe^3^ maun^3^ ja^3^
37	*Upeneus guttatus*	nga^3^ phoun^3^ ji^3^, auʔ mouʔ seiʔ, nga^3^ cɔ^2^ she^2^, (bəjwan^2^ jwan^2^)

Column 1 gives the number of people (from a total of 90 respondents) who provided at least one of the names listed in Column 3. Counts are provided for the Core species only (see Methods for more details)

*N.B.* The first name in each cell of Column 3 is the most commonly recorded Rakhine name. Names in parentheses indicate ethnotaxon labels that were recorded infrequently; they may be from a different dialect, or in rare cases, may represent an incorrect identification. Square brackets indicate an optional name element. < followed by an English word indicates a loanword in the name

*Pronunciation note* A modified (simplified) version of the International Phonetic Alphabet has been used here to maximise precision and intelligibility to a wider audience. *ə*, schwa, as in the ‘a’ of ‘about’; *ɔ*, as in the ‘au’ of ‘caught’; ɛ, as in the ‘e’ of ‘get; *ʔ*, glottal stop; *t̪* and *d̪* indicate dental consonants; *hs*, ‘hissy s’ [s^h^]; *hl*, *hm*, *hn* and *hr* are devoiced *l* [l̥], *m* [m̥], *n* [n̥] and *r* [r̥], respectively; *ph*, *th* and *kh* represent the aspirated stops [p^h^], [t^h^] and [k^h^], respectively; *sh* and *ch* are pronounced as in English; *ng*, velar nasal [ŋ]; *c* is the affricate [tɕ]; *n* at the end of a syllable (*e.g. oun*) indicates a nasal vowel; and superscripts ^1^ ‘creaky’ tone, ^2^ ‘low’ tone, ^3^ ‘high’ tone

It is more difficult to make generalisations about the Rakhine fish name dataset, compared to the Intha names, due to the large number of species involved. However, there seems to be a tendency for pelagic species (such as *Selar crumenophthalmus*) and species associated with coral reefs (*Sargocentron praslin*) and deep water (*Antigonia emanuela, Argyrops spinifer*, *Hapalogenys merguiensis*) to be less well known than nearshore, intertidal or brackish water species (examples of well-known fish that meet the latter criteria include *Eleutheronema tetradactylum*, *Lates calcarifer*, *Scatophagus argus*). As expected, Rakhine people largely could not identify predatory reef-associated fish that are commonly consumed in other parts of the world, such as snappers (*Lutjanus* spp.) and emperors (*Lethrinus* spp.).

A noteworthy feature of the Rakhine dataset is the large number of synonyms for many fish ethnotaxa/species. It is not uncommon for a species to be labelled with four or five names, and some have as many as eight distinct names. Most of the names presented in Table 6 are very likely correct, as the correspondences between pairs or sets of names were stated explicitly during interviews by (often multiple) expert fishermen. For instance, a 49 y.o. fisherman from Kyaukpyauk said that the Indo-Pacific king mackerel *Scomberomorus guttatus* was called *ngənyo*^*1*^* loun*^*3*^ in his village, but was referred to as *ngəshwan*^*3*^ in town (i.e. Kyaukphyu). Both claims were verified through interviews with other fishermen from the two locations. Other forms of naming variation include simple phonological variation, semantic variation, where different aspects of the fish are referred to in the name, and internal lexical variation, where two variant forms of a fish name have the same meaning, but use different lexemes or morphemes. The sociolinguistic aspects of fish nomenclature are highly interesting but complex and will be explored in detail in a future publication.

### Patterns of ethnoichthyological lexical knowledge

Following are the results of nonparametric statistical analyses on the responses from the speakers of the two languages (the data were not normally distributed and frequently had unequal variances.) The dependent variable Total (the total number of stimuli named by each consultant) is only mentioned in case of a significant result.

### Intha

**Fig. 2:**
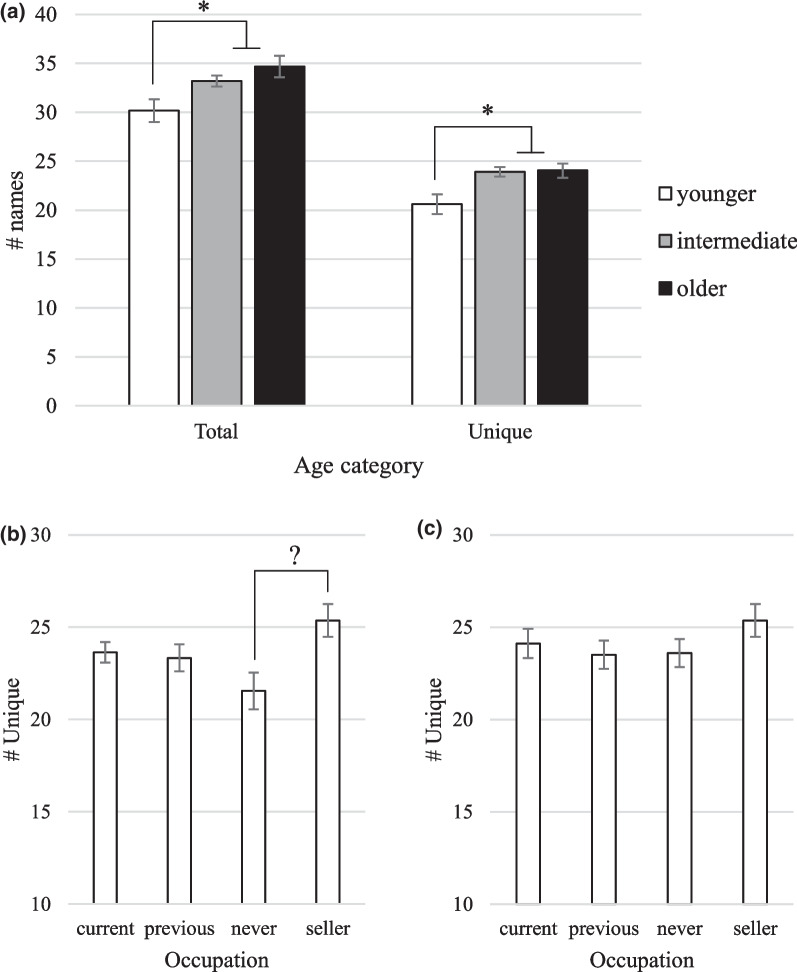
**a** Mean Unique and Total responses according to Age category (Intha). Y-axis shows average number of responses (names) ± S.E; **b**, **c** Mean Unique responses according to the Occupation category (Intha). **b** all respondents; **c** ‘younger’ respondents excluded

A Kruskal–Wallis test on the three Age categories returned a significant result for the dependent variable Unique (*H* = 8.43, *d.f*. = 2, *p* = 0.01), while post hoc pairwise comparisons revealed that it was the ‘younger’ age group that was able to produce significantly fewer Unique fish names than the other two groups (Fig. 2a). The variable Total was also significant, (*H* = 11.05, *d.f.* = 2, *p* < 0.01), with the performance of the ‘younger’ group being significantly worse than that of the ‘older’ group.

Overall, the variable Occupation did not have a significant effect on Unique responses (Kruskal–Wallis *H* = 6.72, *d.f.* = 3, *p* = 0.08). There is some indication in Fig. 2b that sellers might outperform men who have never fished, but this is possibly due to the fact that there were no ‘younger’ respondents among the sellers (the mean age for sellers was 44.36 y.o., compared to 37.7 for the men who have never fished). Excluding younger respondents from the analysis reduces the differences in Unique names among the Occupation categories (Fig. 2c).

An in-depth analysis of possible interactions between Age and Occupation could not be carried out for Intha, due to the small number of sellers, and the very unequal age distributions for both sellers and fishermen. However, it was possible to combine the Unique responses of current and previous fishermen, to see if the categorical Age variable had an effect on people with at least some fishing experience. While there was a very slight trend for Unique responses to increase with increasing age (Table 5), the differences were not statistically significant (Kruskal–Wallis *H* = 1.04, *d.f.* = 2, *p* = 0.59).

**Table 5:** Unique responses for previous and current fishermen combined (Intha)

	Mean	± S.E
Younger	22.62	0.71
Intermediate	23.50	0.72
Older	24.05	0.90

The variable Zone did not have an overall effect on consultants’ responses (Kruskal–Wallis *H* = 4.80, *d.f*. = 2, *p* = 0.09). However, a significant difference did exist in a subset of the data, but this needs to be verified with a larger sample: when regarding just those respondents who have some fishing experience (i.e. current and previous fishermen), the Unique responses from the ‘north’ zone were significantly higher than those from the ‘central’ and ‘south’ zones combined (Kruskal–Wallis *H* = 4.08, *d.f.* = 1, *p* = 0.04). The difference was marginal, however (Table 6).

**Table 6:** Unique responses for respondents from northern villages versus respondents from central and southern villages combined (Intha)

	Mean	± S.E
N	24.35	0.70
C + S	22.91	0.60

Finally, the responses of male and female individuals from the ‘never fished’ category were compared, to determine if there were gender effects in fish knowledge. The dataset was unfortunately small (11 females, 9 males), and although females on average knew more Unique names (f: 23, m: 19.7), there was no statistically significant difference (Mann–Whitney *U* = 32, *p* > 0.05).

### Rakhine

**Fig. 3:**
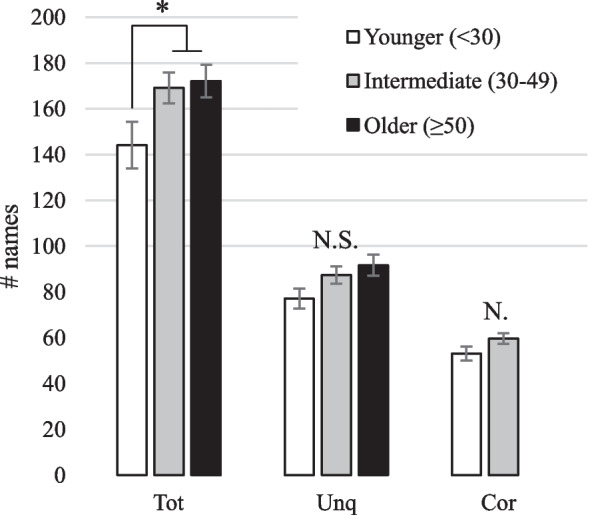
Mean Total, Unique and Core responses according to Age category for all respondents (Rakhine). N.S., not significant

As with the Intha data, the categorical Age variable was found to have a significant effect on the Total number of fish species named by Rakhine speakers (Kruskal–Wallis *H* = 6.06, *d.f*. = 2, *p* = 0.048) (Fig. 3). However, the p value indicates that this effect is marginal, possibly due to the high level of inter-individual variation. Age did not have a significant effect on the dependent variables Unique (Kruskal–Wallis *H* = 5.19, *d.f*. = 2, *p* = 0.07) and Core (Kruskal–Wallis *H* = 4.39, *d.f*. = 2, *p* = 0.11).

**Fig. 4:**
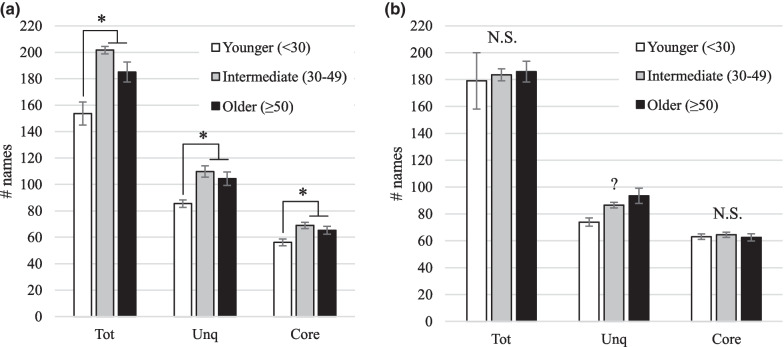
**a** Fishermen only: Mean Total, Unique and Core responses according to Age category (Rakhine). **b** Sellers only: Mean Total, Unique and Core responses according to Age category (Rakhine). N.S., not significant

Next, the effect of Age on the responses of the two largest occupation categories was analysed separately. Looking at just the data from the fishermen, the categorical Age variable was found to have a significant effect on all three dependent variables: Total (Kruskal–Wallis *H* = 14.44, *d.f*. = 2, *p* < 0.001), Unique (Kruskal–Wallis *H* = 16.92, *d.f*. = 2, *p* < 0.001) and Core (Kruskal–Wallis *H* = 10.48, *d.f*. = 2, *p* < 0.01). In all cases, it was the younger fishermen who performed worse than the intermediate and older fishermen (Fig. 4a). The responses of the sellers showed a completely different pattern, as Age category did not have a significant effect on any of the dependent variables (Total, Kruskal–Wallis *H* = 0.412, *d.f*. = 2, *p* = 0.81; Unique, Kruskal–Wallis *H* = 4.723, *d.f*. = 2, *p* = 0.09; Core, Kruskal–Wallis *H* = 0.50, *d.f*. = 2, *p* = 0.77). However, the Unique scores for the sellers did show an increasing trend with Age (Fig. 4b), and so a correlation analysis between age as a continuous variable and the Unique responses for sellers was carried out. The result turned out to be significant (Kendall’s Tau = 0.40, *p* < 0.01). It is also noteworthy that sellers of all age categories had nearly identical responses for the Core fish names (Fig. 4b).

**Fig. 5:**
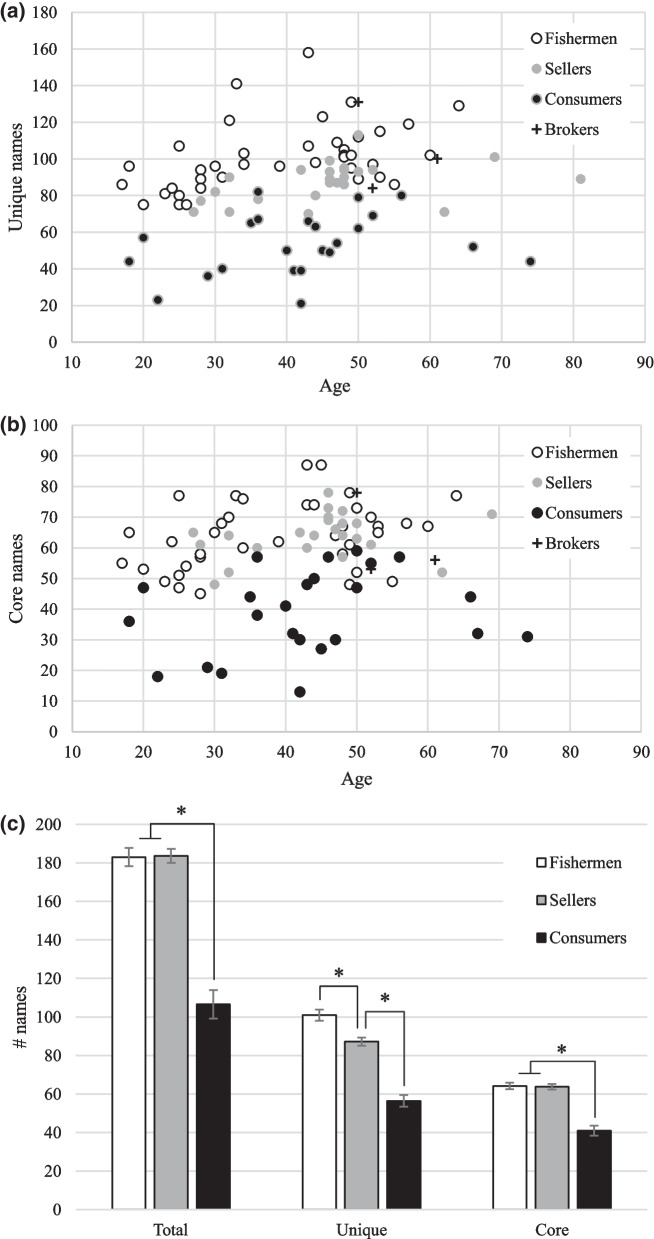
**a** Distribution of Unique responses based on Occupation (Rakhine). **b** Distribution of Core responses based on Occupation (Rakhine). **c** Mean Total, Unique and Core responses based on Occupation (Rakhine)]

The Occupation variable was found to have a significant effect on Rakhine people’s responses, for all three dependent variables measured: Total, Kruskal–Wallis *H* = 39.84, *d.f.* = 2, *p* < 0.001; Unique, Kruskal–Wallis *H* = 48.73, *d.f.* = 2, *p* < 0.001; Core, Kruskal–Wallis *H* = 37.44, *d.f.* = 2, *p* < 0.001. A graphical representation of the combined responses for the Unique names can be seen in Fig. 5. In the case of Total and Core, respondents in the consumer category performed significantly worse than the fishermen and sellers, while the latter two groups were statistically similar (Fig. 5c). The Unique responses showed a different pattern, as the sellers’ performance fell between that of the fishermen and the consumers (Fig. 5). In other words, fishermen were able to produce significantly more Unique names than sellers. The brokers’ data were excluded from this and the previous analysis, but their performance was generally similar to that of the fishermen (the brokers’ data can be seen in Fig. 5).

**Fig. 6:**
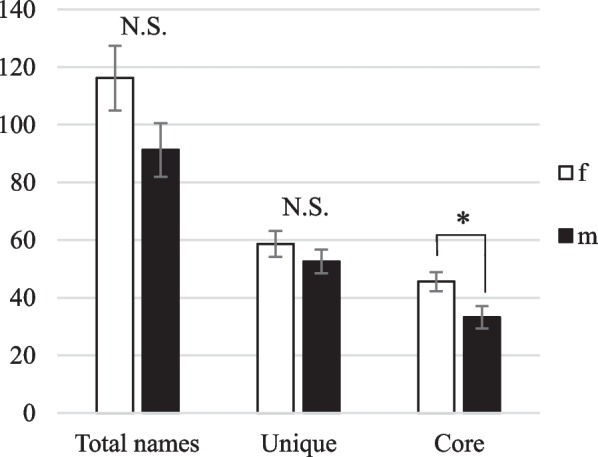
Mean Total, Unique and Core responses based on gender among the consumer group (Rakhine)

Finally, there were gender differences among the group of consumers, *i.e.* people with no direct connection to fishing or the fish trade. Both males and females volunteered similar numbers of Total identifications (Mann–Whitney *U* = 40, *p* > 0.05) and knew roughly similar numbers of Unique names (Mann–Whitney *U* = 47.5, *p* > 0.05), but female respondents performed significantly better at identifying species from the Core group (Mann–Whitney *U* = 11, *p* < 0.05) (Fig. 6). On average, female consumers were able to correctly identify 12 more culturally and/or commercially important fish ethnospecies than male consumers.

## Discussion

The fish name lists collected for both Intha and Rakhine showed that while local knowledge of fish is strong in both communities, there are noteworthy differences in the level to which different fish species can be recognised and named. Among the Intha in particular, ecological changes in Inle Lake seem to be responsible for people being unable to name a handful of small fish species; this is further discussed below in the context of the quantitative results. The results of the statistical analyses show that there are clear differences in the distribution of ethnoichthyological lexical knowledge within each of the two language communities investigated. Moreover, there are also interesting differences between the Intha and Rakhine data, which give some indication of the diverse mechanisms affecting lexical knowledge transmission within each community. In the following discussion, the results from the two communities will be compared, in order to highlight the key similarities and differences in knowledge distribution.

The fish knowledge of both Intha and Rakhine speakers was affected by the variable Age, as has been reported for various kinds of TEK from around the world (e.g. [31–34]). However, there were subtle differences, between the two communities, in the way fish naming ability was affected by age. First, the effect of age at the whole community level was much weaker (in fact, statistically marginal) for Rakhine speakers, and was only evident in the total number of fish that respondents attempted to name. There was a stronger effect among Intha speakers, and younger people (up to the age of 29 y.o.) were able to provide significantly fewer Total and Unique fish names. However, it is difficult to reconcile the above finding with the result that there was no significant effect of Age on the responses of previous or current Intha fishermen. A count of the Occupation groups represented within the three Age categories shows that the ‘younger’ group was dominated by current fishermen and people who had never fished, whereas the ‘older’ group was dominated by previous fishermen. The ‘intermediate’ category contained roughly equal numbers of all four Occupation groups. This suggests that younger people, especially those who have never fished, are able to name fewer fish because they have not been exposed to a number of species that are now rare. It is also possible that the small number of total species to be learnt masks any shallow learning curves that may only be evident in children and young adults. More data (such as fish name elicitations with Intha speakers below the age of 18 y.o.) are required to properly investigate the effect of age, but certain aspects of the Intha name inventory in (Table 5) suggest the following scenario.

There have been reports of declines in fish biodiversity in Inle Lake in recent decades, due to factors such as reduced rainfall, sedimentation, agricultural runoff, sewage from hotels and competition from introduced species like the *T. niloticus* (e.g. [15, 17]. Furthermore, anecdotal evidence from Intha respondents suggests that the illicit practice of electrofishing is responsible for the indiscriminate destruction of fingerlings from target and non-target species. A number of endemic species are now rare [35], while the catches of commercially important species have been decreasing [18]. These reports are corroborated by the finding, in the present study, that very few Intha speakers were able to name the numerous small, often endemic, fish species that Annandale, at the start of the twentieth century, had characterised as being highly abundant all over the lake. As noted above, four small species, including the endemic eel *C. caudata*, could not be identified by a single person. The fact that these are small fish that may be easily missed is not relevant here, as Intha fishermen regularly catch, dry and sell small fish of all varieties, and have done so for generations. In fact, it is due to this practice that the phenomenon of ‘fishing down the food web’, reported from a North American lake following the introduction of an invasive species [36], probably cannot be said to apply to Inle Lake. A likely explanation for our interview results is that the small fish mentioned above are now extremely rare in Inle Lake, or possibly even locally extinct, with their range restricted to nearby shallow creeks and ponds. One elderly respondent, who had worked in the local fisheries office, even stated categorically that the fish called *nga*^*3*^* taun*^*2*^* nwɛ*^*2*^ (listed in Annandale as *Barbus compressiformis*, but now *Systomus compressiformis*) was no longer present in the lake. Note that this fish was sighted during a 1997 survey by Su and Jassby [17], but not in Kano et al.'s [20] 2014 and 2016 surveys. It is currently listed as “critically endangered (possibly extinct)” in the International Union for Conservation of Nature (IUCN) Red List [37]. This species did not form part of the Intha stimulus set in the present study, as no photograph could be sourced online.

**Fig. 7:**
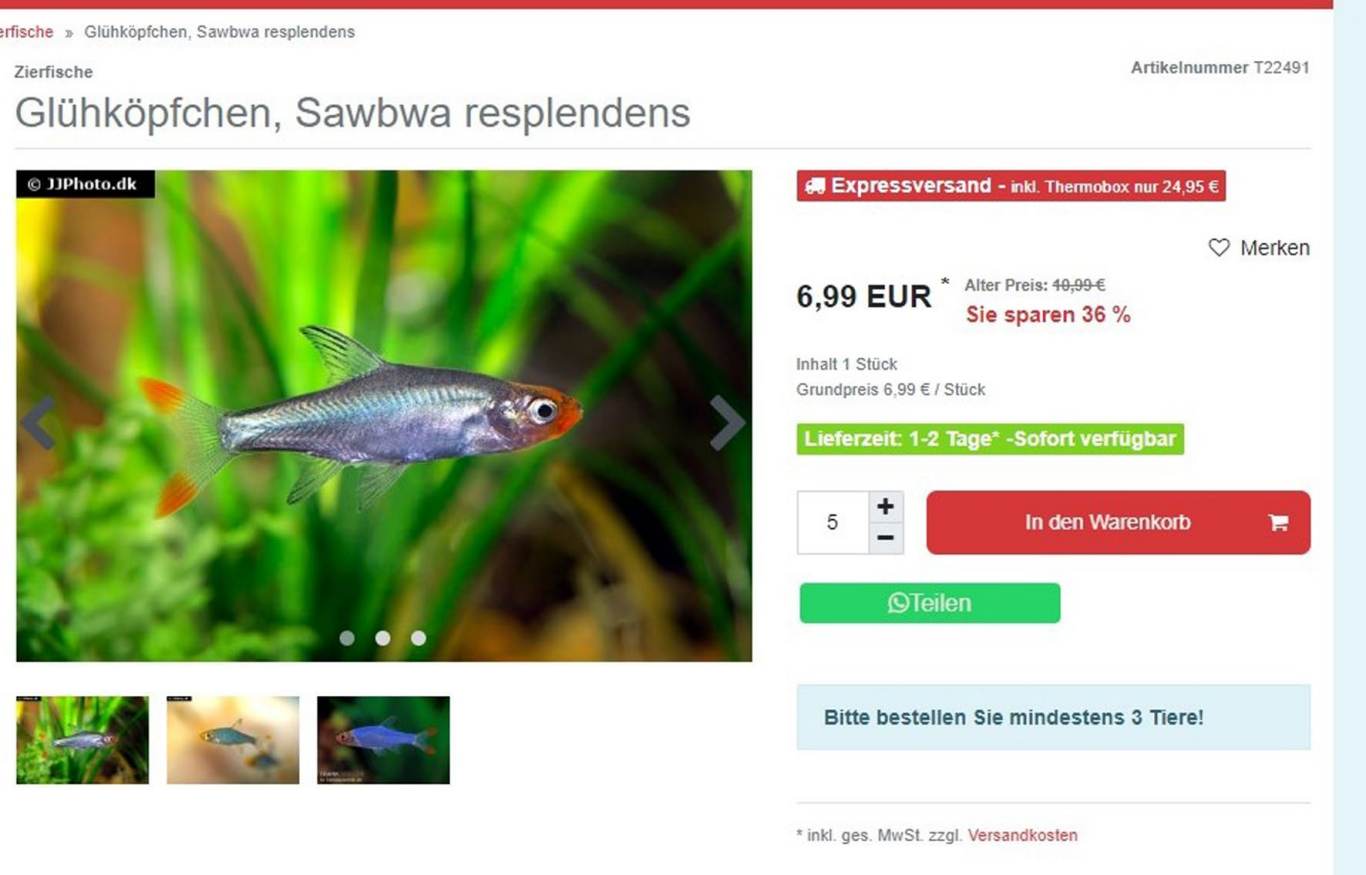
Live (presumably captive-bred) *Sawbwa resplendens* available for sale on a German aquarist website

The example of the beautiful endemic fish *S. resplendens* is informative of the age-related differences in fish TEK among Intha people, and of some of the factors that may be responsible for these differences. While live specimens were caught by Kano et al. [20] in Shan State, the location data provided by the authors on the Database for Freshwater Fish (https://ffish.asia) suggest that the fish currently occur only in small, sometimes artificial, ponds and shallow streams in the vicinity of Inle Lake, and not in the lake proper. It is therefore unsurprising that only around 20% of Intha respondents were able to identify this fish correctly. Of these 18 individuals, the two youngest were in their 30 s (33 and 37 y.o.), three others were in their 40 s, and the rest were aged 50 years or older. *S. resplendens* has long been sought after as an ornamental fish for the aquarium trade, along with other local species, such as *G. gravelyi* and *M. rubescens*, and there is anecdotal evidence of live specimens of these species being collected from the lake by ‘Gurkha’ people in the 1990s. Nowadays, live *S. resplendens* can be purchased online even in European countries (Fig. 7), and it is ironic that it is almost unknown in that part of the world where it is supposed to be endemic.

As mentioned above, there was only a weak overall effect of Age on Total fish names in Rakhine. However, far stronger Age effects are seen when data from fishermen only are analysed. This reveals that the younger fishermen are outperformed by their counterparts in the other two Age categories, which is likely a reflection of the steep learning curve the former face when starting their fishing careers. The data indicate that by the age of 30 y.o., a Rakhine fisherman’s knowledge will have plateaued at an average of 110 fish names (at least with respect to the 218 species investigated in the present study). Looking at the data from the sellers only, it appears that Unique names—presumably those labelling rarer or more unusual fish species—continue to be learnt until later in life, as reflected in the positive correlation between age as a continuous variable and Unique names. Among the sellers, the Core name responses are virtually identical for all three Age categories, indicating that these culturally and/or commercially important ethnotaxa are learned early in a young woman’s market career. In contrast, young fishermen know fewer Core names than their older counterparts, as they do not actively buy fish at the market and therefore need time to reach the expertise of older fishermen.

Overall, there was no effect of Zone on the Intha data, other than that men from northern villages with at least some fishing experience knew a few more Unique names than similar men from the central and southern villages combined. This finding may be the result of more intense non-fishing activity in the central and southern villages (farming and weaving, respectively), while the northern zone villages tend to focus solely on fishing. The difference is marginal, however, and a repeat analysis with a larger sample (perhaps focusing on younger males) will help clarify the issue.

Intha and Rakhine differed greatly in the responses provided by people involved in different Occupations. Occupation had no significant effect on Unique fish scores in Intha, but in Rakhine, there were stark differences to be seen, particularly in terms of the generally high performances from fishermen and sellers on the one hand, and the generally poor performance of the consumers on the other. It was noted that Rakhine consumers did not even attempt to identify close to half of the stimulus pictures (as indicated by the low Total score), whereas fishermen and sellers tried to name almost all. On average, consumers only knew approximately two thirds of the Core fish identified by the fishermen and sellers. This is probably because any given Rakhine household tends to consume a fixed inventory of fish species. Households may differ from one another in the fish they consume, but in general, consumers tend to be quite neophobic when it comes to trying out unknown fish species. The problem is exacerbated by the fact that certain fish species (such as the Asian sea bass *Lates calcarifer*, the silver pomfret *Pampus argenteus* and the Indian threadfin *Leptomelanosoma indicum*) are highly prized, while many others may be considered inferior (for a range of idiosyncratic reasons, such as having a strong odour, being too big, too bony, too colourful, too cheap or simply not tasting ‘good’). Socio-economic status may also play an important role in fish choice: a middle-class urban household might avoid species such as the spotted scat *Scatophagus argus*, all sharks, various reef fish of the families Acanthuridae, Balistidae, Chaetodontidae, Pomacanthidae, Siganidae, groupers *Epinephelus* spp. and queenfishes *Scomberoides* spp., but these might be consumed sporadically in poorer households. As a result, the average consumer is exposed to far fewer fish species than are regularly sold at market.

It is interesting to note that the ‘consumer’ category in the Rakhine dataset is comparable to the ‘never fished’ category in the Intha dataset, and yet, individuals from the two categories present very differently when compared to individuals from the other Occupation categories in their respective communities. Rakhine consumers, as noted above, were significantly outperformed by fishermen and sellers, whereas Intha individuals who had never fished seemed to be more or less as knowledgeable as the fishermen and sellers in their community. It is possible that the smaller Intha fish inventory allows even non-specialists to eventually reach the levels of expertise possessed by professionals in the fish trade. In Rakhine, however, the enormous species diversity, coupled with strong dietary biases among the local populace ensure that only professionals become familiar with a large number of fish species. Among the professionals too, Rakhine fishermen and sellers differed in the number of Unique names volunteered. (As noted above, there was no difference among the Intha.) This may also be a result of high species number, as well as the fact that a handful of fish types may be considered unmarketable and discarded at sea by fishermen. It should be noted that in the Kyaukphyu artisanal fishery, ‘there is no fish bycatch’ [22], and that even those fish species that are not consumed by humans are kept, dried, and turned into animal feed. However, since the sellers in the local market only handle fish that are to be eaten by people, it is likely that they simply do not encounter a number of the unusual or unpalatable species that fishermen are familiar with. This also explains why sellers knew, on average, a similar number of Core fish names when compared to fishermen. Brokers, on the other hand, appear to know as much as fishermen, as they have to deal with all fish types.

The comparison by Occupation of fishermen and sellers can also be regarded as a de facto investigation of gender effects on fish naming ability. Previous studies have shown that gender can have an impact on access to fishery resources and catch volumes [27, 38], as well as on individuals’ knowledge of local biodiversity Renck et al. [12]. The latter study, on an artisanal fishing community in north-eastern Brazil, showed that men tended to be able to name more fish species than women. However, the women interviewed in the above studies also engaged in fishing activities, and are therefore not fully comparable to the women in the present study. The present study also tested the effect of gender on small datasets of ‘lay’ people both communities, *i.e.* Intha people who had never fished and Rakhine consumers of fish. Among the Intha, there was no significant difference between males and females who had never fished, although there was a tendency for females to outperform the males. Repeating the analysis with a larger dataset may yield a significant result in favour of females. For Rakhine, however, female consumers were found to be significantly better at identifying Core fish species than male consumers. In the present study, Rakhine males knew fewer Core fish in spite of knowing as many Unique fish names as females; this suggests a general unfamiliarity with culturally important fish ethnotaxa among males, even if the names are heard every now and then in daily conversation.

A probable reason for females performing better is that they tend to be the ones who go shopping for food at the local market. Despite the average Rakhine consumer’s reluctance to try out new fish (or possibly as a result of it), it is common to see sellers at the market encouraging potential customers to buy an unknown fish variety by mentioning its name, extolling its culinary properties, pointing out similarities with other common species, and even providing suggestions on how to cook it. Even if the consumer remains unconvinced, it is easy to see how a woman who regularly goes to the fish market will eventually be able to identify some fish that she has never eaten. The limited knowledge of fish consumers seems to be a general problem worldwide, as is fish sellers’ willingness to take advantage of this phenomenon [39]. In Kyaukphyu markets, adulteration of expensive fish species with cheaper types often occurs (e.g. Asian sea bass *La. calcarifer* or Indian threadfin *Le. indicum* with blackmouth croaker *Atrobucca nibe*), mostly in the context of the sale of pre-chopped fish (the fish is sold as transverse slices including the backbone, rather than the boneless sagittal fillets seen in Western supermarkets.)

## Conclusions

Fish species are recognised to varying degrees in both communities for different reasons: for the Intha, the abundance or rarity of a species appears to be responsible, whereas for Rakhine people, the usual habitat of a fish and/or its palatability seems to be the key factors. Fish TEK is distributed in different ways in the two communities in terms of the age, gender and occupation of individuals. Whether the small species inventory available to Intha people is responsible for a more homogenous distribution of TEK in that community remains to be determined through further research. Ecological disturbances leading to the local extinction of many fish species may explain why younger Intha knew fewer fish names than their older counterparts. On the other hand, the various differences observed for Rakhine in terms of age, occupation and gender can be explained by the experience required to learn a large number of fish species, and the way Rakhine people interact with fish on a day-to-day basis. Household-level preferences for certain fish species may also play an important role in determining which fish are known to which people. The present study provides further support for the need to regard TEK as a variable phenomenon, and to make intra-community variation a key object of investigation. It also highlights the advantages of teasing apart people’s interactions with key species, even in the context of a research programme that encompasses an entire ethnobiological domain.

## Data Availability

The datasets used and/or analysed during the current study are available from the corresponding author on reasonable request.

## Abbreviations

FAO: Food and Agricultural Organization
IUCN: International Union for Conservation of Nature
TEK: Traditional ecological knowledge
